# Staging classification of atypical ulnar fractures

**DOI:** 10.1007/s11657-025-01569-6

**Published:** 2025-06-19

**Authors:** Tetsushi Kinoshita, Fumihiro Isobe, Hiroshi Yamazaki, Koichi Nakamura, Shun Hashimoto, Teruki Shirayama, Hiroko Iwakawa, Masanori Hayashi, Jun Takahashi

**Affiliations:** 1 Department of Orthopaedic Surgery, Shinshu University School of Medicine, Matsumoto, Japan; 2 Department of Orthopaedic Surgery, North Alps Medical Center Azumi Hospital, Ikeda-machi, Japan; 3 Department of Orthopaedic Surgery, Aizawa Hospital, Matsumoto, Japan; 4 Department of Orthopaedic Surgery, Nagano Municipal Hospital, Nagano, Japan

**Keywords:** Atypical ulnar fracture, Bisphosphonates, Osteoporosis, Autologous bone grafting

## Abstract

***Summary*:**

Atypical ulnar fractures (AUFs) are rare fractures associated with long-term bisphosphonate use; their progression pattern is not understood. This study classified AUFs based on imaging characteristics, revealing a progression from cortical bone thickening to complete fractures with osteosclerosis over time. This classification may assist in determining appropriate treatments for AUFs.

**Purpose:**

The progression and characteristics of AUFs remain unclear, and no definitive treatment method has been established. This study aimed to classify AUFs based on imaging findings to elucidate their characteristic progression patterns.

**Methods:**

We retrospectively reviewed 12 AUFs in 11 patients who had used bisphosphonates in the long term. Based on imaging characteristics, we classified the fractures into 3 stages: Stage I (incomplete fractures with cortical thickening or fracture lines only in the dorsal cortex), Stage II (complete fractures with fracture lines extending to the ventral cortex), and Stage III (complete fractures with osteosclerosis).

**Results:**

At the initial examination, 2 AUFs were classified as Stage I, 8 as Stage II, and 2 as Stage III. Two fractures progressed from Stage I to Stage II during follow-up. Our imaging analysis showed a consistent pattern, suggesting that AUFs begin with cortical thickening in the dorsal cortex, progress to fractures extending to the ventral cortex, and develop sclerosis resembling pseudoarthrosis.

**Conclusion:**

AUFs begin with cortical bone thickening and progress to complete fractures. Over time, these complete fractures can become sclerotic, resembling pseudoarthrosis.

## Introduction

Atypical ulnar fractures (AUFs) are fractures associated with prior long-term use of bisphosphonates (BPs). BPs are among the main drugs used for the treatment of osteoporosis; however, BPs have been shown to inhibit the normal repair of microdamage due to marked suppression of bone turnover. Consequently, there is an increased risk of fractures [1, 2]. Fractures that occur via this mechanism are called “atypical fractures.” In previous reports, atypical fractures are often associated with subtrochanteric femoral fractures and femoral shaft fractures [1, 2]. AUFs are known to occur in rare cases [3–5]. As the number of osteoporosis patients increases in the future, the number of atypical fractures, including AUFs, is expected to increase.

Past reports have indicated that sclerotic lesions at fracture sites in AUFs are characterized by increased osteoid width and a lack of osteoclasts, suggesting that these fractures are associated with severely suppressed bone turnover [6]. As a result, atypical fractures, including AUF, have a prolonged healing period [7, 8] and a higher risk of nonunion with conservative treatment [9]. Despite these insights, the mechanisms underlying the progression of AUFs remain unclear, and a consensus on optimal treatment strategies has yet to be established. Staging AUF based on imaging findings allows evaluation of AUF using a unified scale and can contribute to the establishment of treatment strategies. In this study, we surveyed 12 AUFs of 11 patients who developed fractures after long-term use of BPs. We classified AUFs into 3 stages based on their imaging characteristics. In addition, we investigated and examined the treatment methods and postoperative outcomes for each stage.

## Methods

This multicenter retrospective study involved 6 hospitals with hand surgeons. Between April 2013 and June 2023, a list of patients was compiled from those diagnosed with ulnar fractures. From this list, patients younger than 18 years and those with open fractures were excluded. The method of diagnosing AUF was based on a report by Tan et al. [10] and included ulnar fractures in the proximal third or the junction between the proximal and middle thirds, transverse fractures, and cortical thickening in the surrounding bone.

**Fig. 1:**
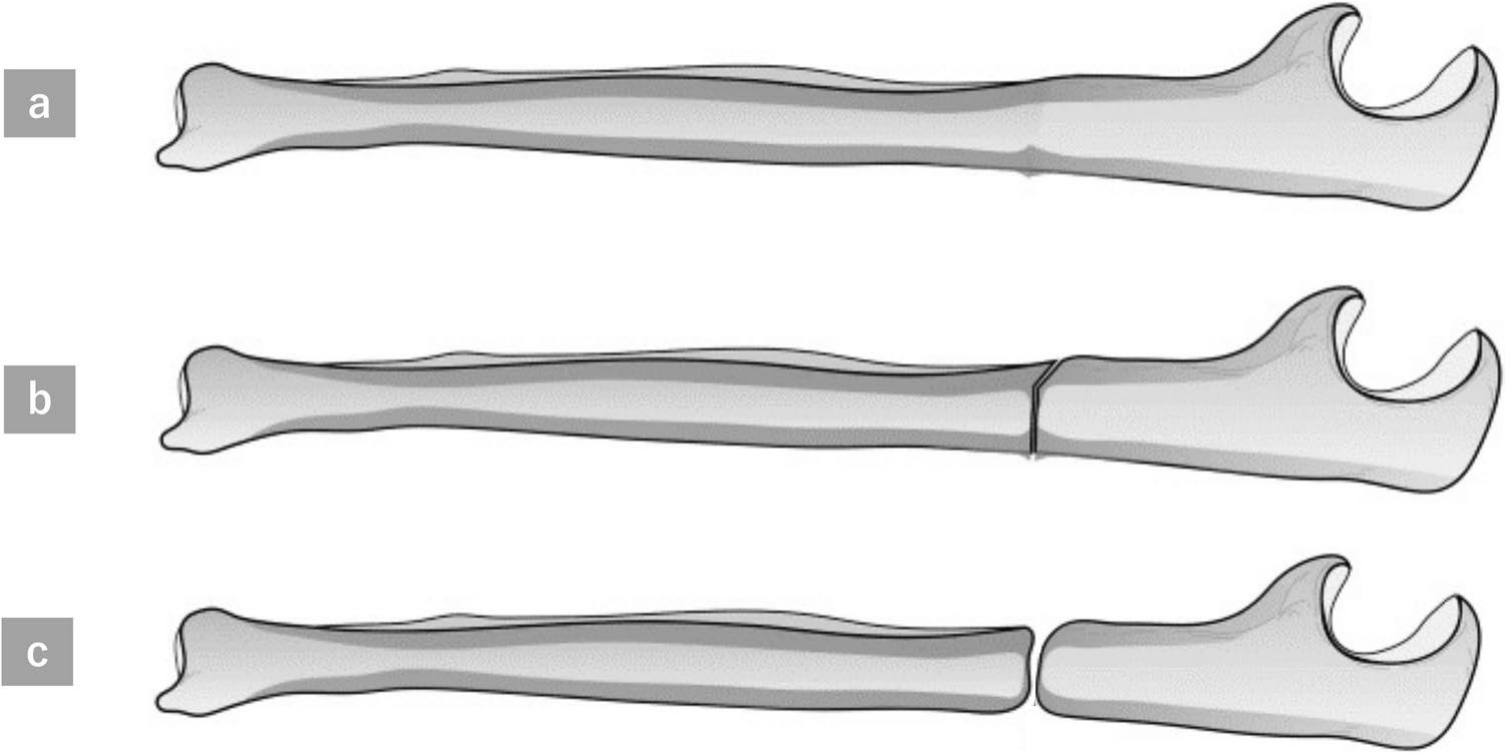
Progression of atypical ulnar fractures. Stage I: Incomplete fracture with thickening or fracture lines only in the dorsal cortex (**a**). Stage II: Complete fracture with fracture lines reaching the ventral cortex (**b**). Stage III: Complete fracture with osteosclerosis (**c**)

The AUFs were classified into 3 stages based on imaging characteristics of lateral forearm radiographs: incomplete fractures with thickening or fracture lines only in the dorsal cortex (Stage I), complete fractures with fracture lines reaching the ventral cortex (Stage II), and complete fractures with osteosclerosis (Stage III) as described in Fig. 1. We investigated and examined the fracture stage at the time of initial examination, distance from the olecranon to the fracture site, treatment method, implants used, use of postoperative parathyroid hormone (PTH) preparations, bony union, and complications/reoperation. Bony union was defined as the bridging callus or the absence of fracture line in the palmar and dorsal cortices on the lateral forearm radiographs.

## Results

**Fig. 2:**
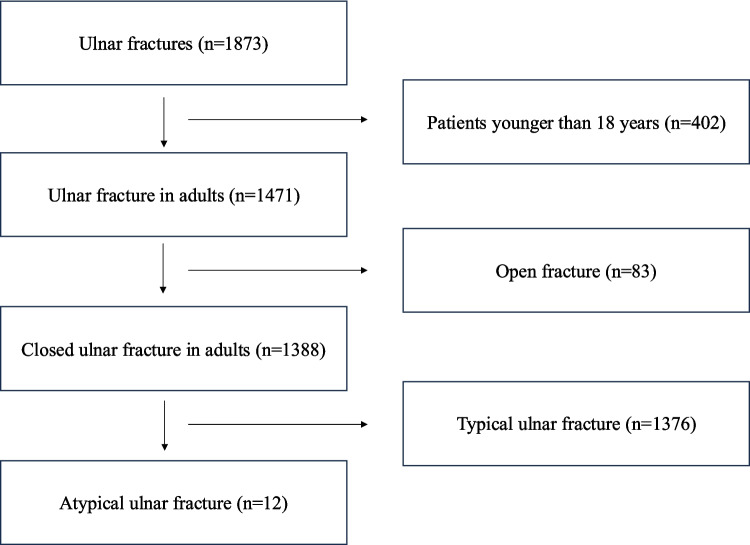
Flow diagram of the patient cohort selection process

Among 1873 cases of ulnar fractures, AUFs were observed in 11 cases and 12 ulnas (Fig. 2). All patients were females, and the mean age at onset was 78.5 years (range 37–90 years). Surgery was performed in 11 AUFs, except for 1 AUF that underwent conservative therapy. Ten out of 11 patients required walking aids before the injury: 3 used a walking stick, 4 used a walking frame, and 3 used a wheelchair. Four patients had a history of steroid use. Among these patients, 2 had rheumatoid arthritis, 1 had systemic lupus erythematosus, and 1 had nephrotic syndrome. In addition, 7 patients had a history of femoral fracture, 3 of whom had atypical femoral fracture (AFF) (Table 1).

**Table 1:** Patient reports of atypical ulnar fractures

Patient No	1		2: left side		2: right side	3	4		5	6	7	8	9	10	11
	Demographics and clinical characteristics														
Age	37		81			85	90		82	84	84	77	78	85	81
Gender	Female		Female			Female	Female		Female	Female	Female	Female	Female	Female	Female
Co-morbidities	Systemic lupus erythematosus		Rheumatoid arthritis Autoimmune hepatitis			NR	NR		NR	NR	NR	Rheumatoid arthritis	NR	Nephrotic syndrome	NR
History of steroid use	Yes		Yes			No	No		No	No	No	Yes	No	Yes	No
History of femoral fracture	Yes^*^		Yes			No	No		Yes	No	Yes^*^	NR	Yes^*^	Yes	Yes
Ambulatory status	Wheelchair		Wheelchair			Walking stick	Walking stick		Walking frame	Walking frame	Walking frame	Walking frame	Walking stick	Wheelchair	Walking without assistance
T score of lumbar spine	−1.5		−2.6			−0.2	NR		−2.0	−1.7	−1.6	−0.8	NR	NR	−2.7
T score of femoral neck	−1.4 (Left) −2.6^†^ (Right)		NR			−1.1 (Left) −1.1 (Right)	NR		−0.5	−2.4 (Left) −2.1 (Right)	NR	−2.5	NR	NR	−1.8
Duration of using bisphosphonate	13 years		Over 10 years			7 years	Over 10 years		NR	NR	6 years	12 years	10 years	Over 10 years	Over 10 years
	Atypical ulnar fracture														
Stage	II^‡^		II^‡^		I → II	III	III^‡^		II	II	II	II	II	II	I → II
Distance from olecranon to fracture(mm)	77		67		65	76	115		70	76	64	70	85	71	97
Surgery	1st	2nd	1st	2nd	1st	1st	1st	2nd	1st	1st	1st	1st	1st	1st	None
Decortication	No	Yes	Yes	Yes	Yes	Yes	Yes	Yes	No	Yes	No	Yes	No	Yes	-
Bone graft	No	No	No	No	No	No	Yes	Yes	No	No	No	No	No	Yes	-
Implant	SLP	ALP	SLP	ALP	ALP	SLP	ALP	ALP	SLP	SLP	SLP	SLP	ALP	ALP	-
	LCP M^§^ plate	VariAx DF^||^ plate	LCP M^§^ plate	VA-LCP PU^¶^ plate	VA-LCP O^**^ plate	LCP M^§^ plate	VariAx DF^||^ plate	A.L.P.S. elbow O^**^ plate	LCP M^§^ plate	LCP M^§^ plate	LCP M^§^ plate	LCP M^§^ plate	VA-LCP PU^¶^ plate	VA-LCP PU^¶^ plate	-
Plate length(mm)	86	156	99	184	142	125	156	194	86	125	99	99	131	157	-
	Postoperative														
Parathyroid hormone	No	No	No	No	No	Yes	Yes	No	Yes	Yes	Yes	Yes	No	No	-
Peri-implant fracture	Yes	No	Yes	No	No	No	No	No	No	No	No	No	No	No	-
Postoperative loss of reduction	No	No	No	No	No	No	Yes	No	No	No	No	No	No	No	-
Infection	No		Yes		No	No	No		No	No	No	No	No	Yes	-
Outcome (Duration of bony union)	Union (16 months)		Union (9 months)		Union (11 months)	Unknown	Union (6 months)		Union (8 months)	Union (32 months)	Union (7 months)	Union (14 months)	Union (12 months)	Union (13 months)	Union (union time unknown)

*NR:* not recorded in detail, *SLP:* straight locking plate*, ALP:* anatomical locking plate

*atypical femoral fracture, ^†^reference values due to post operation, ^‡^ findings at the time of reoperation are listed in the right column, M^§^ metaphyseal, DF^||^ distal fibula, PU^¶^ proximal ulnar, O^**^olecranon

All 11 patients were using BPs for osteoporosis. In two cases, the duration of use could not be confirmed (Patient No. 5 and 6). The duration of BP use in this study ranged from 6 to 13 years.

**Fig. 3:**
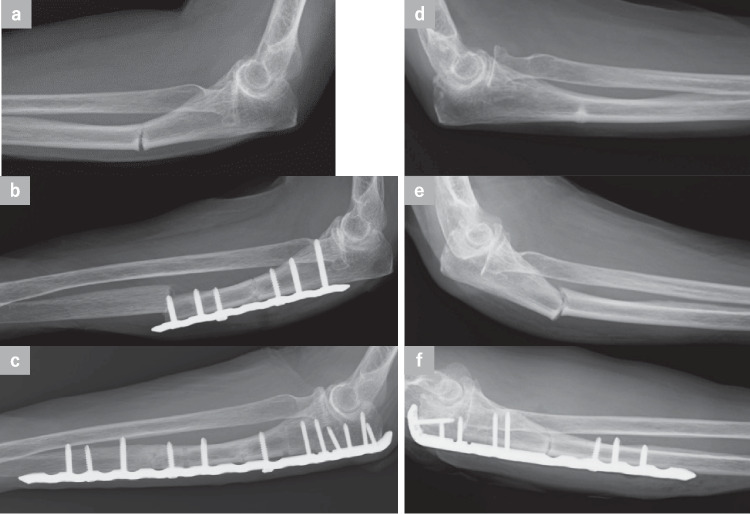
An 81-year-old woman (Patient No. 2) had been taking bisphosphonate (BP) preparations for more than 10 years for osteoporosis treatment. Pain developed in her left forearm after she threw an object. Radiographs revealed a transverse fracture in the proximal third of her left ulna and thickening of the dorsal cortex, indicating Stage II atypical ulnar fracture (AUF) (**a**). She was also diagnosed with a Stage I AUF on the right side (**d**). The right ulna fracture was displaced after 2 weeks and classified as an advanced Stage II AUF (**e**). She underwent open reduction-internal fixation using an anatomical locking plate (ALP) (VA-LCP olecranon plate; DePuy Synthes, Warsaw, IN) (**f**). Three weeks after surgery on the left forearm, a fracture was observed at the most distal point of the plate (**b**). Reoperation was performed by ALP (VA-LCP proximal ulnar plate; DePuy Synthes, Warsaw, IN) (**c**). Bony union was achieved on the left and right sides 8 and 6 months after the final surgery, respectively

**Fig. 4:**
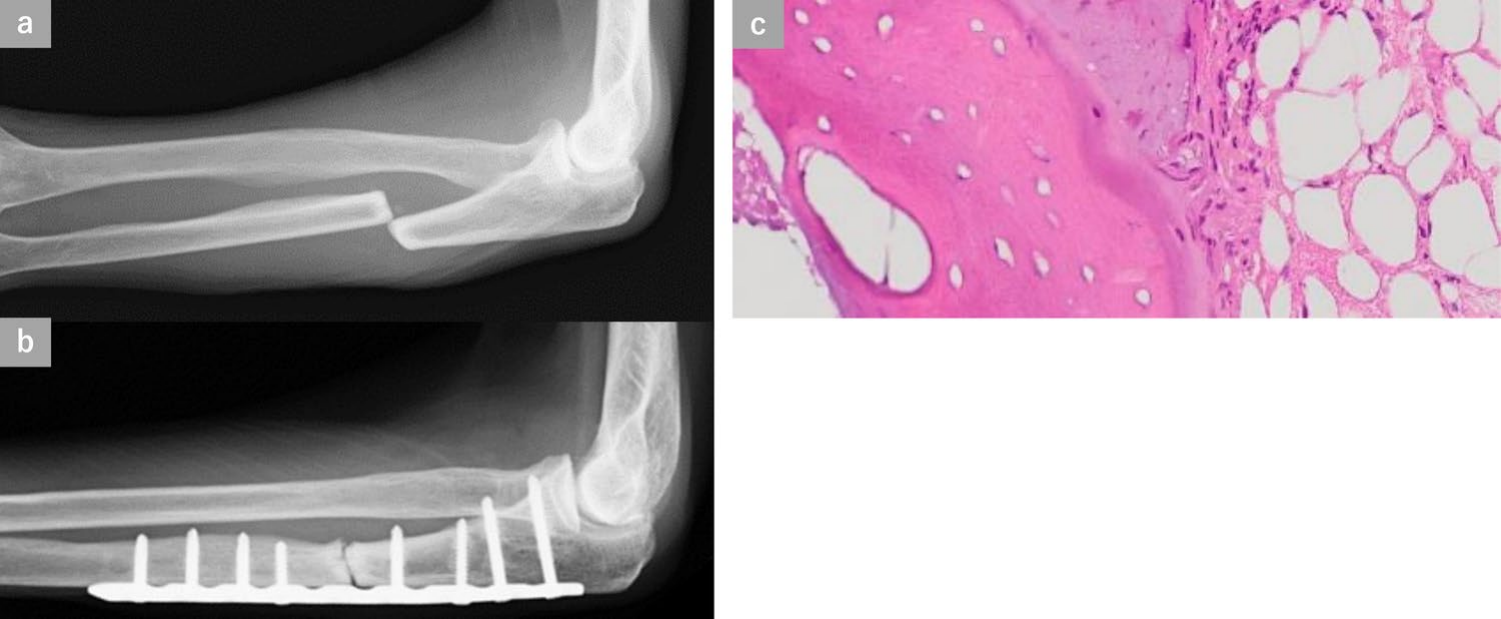
An 85-year-old woman (Patient No. 3) had a history of BP use for 7 years. When she was getting dressed, she noticed pain in her left forearm. Radiography revealed a transverse fracture with displacement of the proximal third of the ulna. The bone cortex around the fracture was thickened, the fracture showed osteosclerotic changes, and the medullary canal was closed, indicating a Stage III AUF (**a**). Surgery was performed using a straight locking plate (SLP) (LCP metaphyseal plate; DePuy Synthes Warsaw, IN) (**b**). Pathological findings of bone tissues collected from the fracture site revealed that there were no osteoblasts or osteoclasts on the surface of the trabecular bone, suggesting the suppression of bone turnover (**c**)

Of the 12 AUFs in 11 cases, 2 had incomplete fractures (Stage I) (Fig. 3d), 8 had complete fractures (Stage II) (Fig. 3a), and 2 had complete fractures with osteosclerosis (Stage III) (Fig. 4a) at initial examination. Two AUFs progressed from Stage I to Stage II after low-energy trauma (Fig. 3e) (Patient No.2,12). One underwent conservative treatment. The exact fusion period is unclear due to a lack of follow-up, but union was confirmed when the patient returned for evaluation 7 years later. All the fractures were transverse fractures, with an average distance of 77.8 mm from the olecranon (range 64–115 mm). Open reduction and plate fixation were performed in 11 AUFs (9 Stage II and 2 Stage III). Decortication was performed on 7 AUFs (5 Stage II and 2 Stage III) by drilling into the fracture site (Patient No.2–4,6,8,10). Resection and curettage of the sclerotic bone were performed on 2 Stage III AUFs for additional procedures. Free autologous bone grafting was performed on 2 AUFs (1 Stage II and 1 Stage III, Patient No.4,10). The mean plate length at the time of the initial surgery was 118.6 mm (range 86–157 mm), and straight locking plates (SLPs) were used in 7 AUFs, whereas anatomical locking plates (ALPs) were used in 4 AUFs. Postoperatively, osteoporosis treatment was changed to the administration of PTH preparations in 6 patients. Postoperative complications included loss of reduction in 1 AUF (Patient No.4) and peri-implant fractures in 2 AUFs (Patient No.1,2). All these complications required reoperation. In all 3 AUFs that underwent revision surgery, the implant was changed to ALP, which allowed for fixation of the proximal bone fragment with 4 or more screws (Patient No.1,2,4). In addition, reoperation was also performed in 2 AUFs that showed postoperative superficial infections (Patient No.2,10). All 10 Stage II AUFs achieved bony union; however, in 1 of the 2 Stage III AUFs, bony union could not be confirmed due to loss to follow-up (Table 1, Patient No.3). Excluding 1 AUF treated conservatively and 1 AUF in which bony union was not confirmed, the average bony union period for the remaining 10 AUF cases was 12.8 months (range 6–32 months).

## Discussion

In this study, we classified AUFs into 3 stages based on the imaging characteristics of lateral forearm radiographs. In addition, we investigated treatment methods and bone union in each case. Nine Stage II and 2 Stage III AUFs initially underwent plate fixation. Free autologous bone grafting was performed only in 2 AUFs (Patient No.4,10). Although 3 AUFs required revision surgery, all AUFs in Stage II and 1 AUF in Stage III achieved bony union. This is the first report to classify AUF based on imaging characteristics. The progression of AUFs appears to share similarities with that of AFFs, which typically begin with cortical bone thickening and progress to complete fractures, sometimes developing sclerotic changes over time and resembling pseudoarthrosis.

We searched for previous AUF case reports that reported plain radiography at the time of fracture and found 12 AUFs in 11 patients (Table 2) [3, 4, 6, 9, 11–16]. In these reports, 10 patients had a history of BP use for 5 years or more. Bauer et al. reported that the risk of developing AFF was 7.29 times higher in patients with a history of BP use for 5 to 7 years compared to those with less than 1 year of use [17]. There have been no specific reports on the relationship between the duration of BP use and fracture risk in the AUF. In this study, AUF patients were more likely to be BP users for more than 6 years. Therefore, as with AFF, long-term BP use appears to be a risk for developing AUF.

**Table 2:** Past reports of atypical ulnar fractures

Author Patient No.	Oh, BH. (2018) 1	Abe, K. (2020) 2	Ito, H. (2019) 3	Osada, R. (2015) 4	Cha, SM. (2021) 5	Moon, J. (2013) 6	Asano, Y. (2020) 7	Murai, A. (2021) 8	Ohta, S. (2022) 9-left side right side		Shimada, Y. (2017) 10 11	
	Demographics and clinical characteristics											
Age	72	84	78	85	70	76	86	74	65		79	89
Gender	Female	Female	Female	Female	Female	Female	Female	Female	Female		Female	Female
Co-morbidities	No	NR	Compression fracture	Compression fracture	NR	Parkinson’s disease	NR	Thyroid cancer	Breast cancer		NR	NR
Duration of using bisphosphonate	7 years	Over 10 years	10 years	7 years	NR	Over 10 years	6 years	Over 10 years	8 years		6 years	9 years
	Atypical ulnar fracture											
Stage	II	II	II	III	II	II	II	I	II	II	III	II
	Operation											
Bone graft	No	Yes	Yes	Yes	NR	NR	Yes	No	Yes	NR	Yes	Yes
Implant	SLP	SLP + ALP	SLP	ALP	SLP	SLP	SLP	SLP	SLP	SLP	SLP	ALP
	Postoperative											
Parathyroid hormone	NR	Yes	Yes	NR	NR	NR	No	No	No	No	Yes	Yes
Postoperative loss of reduction	No	No	Yes	No	NR	No	No	No	Yes	No	No	No
Outcome	Union	Union	Union	Union	Union	Union	Union	Union	Union	NR	Union	Union

*NR* not reported in the original study, *SLP* straight locking plate*, ALP* anatomical locking plate

Stage I AUF, as defined in this study, represents an incomplete fracture. The necessity of prophylactic surgical intervention for incomplete AUFs has not yet been conclusively established. In AFF, the insertion of a preventive intramedullary nail is recommended if insufficiency fractures are high risk based on factors such as the fracture location, nature of pain, and condition of the contralateral femur [18]. In cases within this study that progressed from Stage I  to Stage II (Patient No.2), a complete fracture was observed on the contralateral side, and the location of the incomplete fracture matched that of the complete fracture. This suggests a risk of progression to a complete fracture. As suggested by our findings, complete fractures can sometimes lead to additional complications, such as peri-implant fractures, postoperative loss of reduction, or infections. Therefore, for cases deemed at high risk of progressing to a complete fracture, preventive surgery may be a viable treatment option.

There is no consensus on the necessity of bone grafting for the surgical treatment of AUFs. In previous reports, bone grafting was performed on 5 of 9 Stage II AUFs [3, 9, 11–16]. In our study, bone grafting was performed in 1 of 9 Stage II AUFs that underwent surgery. As the bony union rate in Stage II AUFs seemed similar to that in other reports, we believe that bone grafting is not necessary for the surgical treatment of Stage II AUFs. Stage III AUFs are considered to have developed bone sclerosis at the fracture site due to the passage of time since the injury. The pathological findings of the fracture site in Patient No. 3 and previous reports [6] revealed necrosis of bone cells at the fracture site. In both our case and other reports, resection and curettage of the sclerotic bone and drilling with Kirshner wires at the fracture site were performed for most AUFs. Especially in Stage III AUFs, bone defects were caused by massive resection and curettage of the sclerotic bone. Therefore, bone grafting is considered to bridge the fractured parts by filling the bone defects. We believe that all these procedures are necessary for the surgical treatment of Stage III AUFs as conventional pseudoarthrosis surgery.

Among the patients who underwent reoperation, there were 2 AUFs of peri-implant fractures, both of which were Stage II AUFs. Both AUFs with peri-implant fractures had fractures at the distal end of the plate. This may suggest that a characteristic external force is acting on the ulna after plate fixation. In addition, all of the AUFs that required reoperation were observed in patients who used wheelchairs, and the use of the forearm as a weight-bearing extremity when transferring to and from a wheelchair in the early postoperative period may have potentially caused the peri-implant fracture. Furthermore, as described in previous reports [7, 8], prolonged healing was observed in many patients. Therefore, the patient should have been instructed to avoid loading the upper extremities as much as possible and to wear a brace after surgery. Furthermore, Abe et al. reported the use of double plating for fixation in AUFs [11]. The use of a double plate may be a treatment option in patients whose affected limb is difficult to unload.

This study had several limitations. First, the number of cases was limited. Second, owing to the lack of established treatment methods, the quality of the surgical techniques may have affected the outcomes, such as bone union. Finally, treatment outcomes lacked long-term follow-up data.

## Conclusions

We classified AUFs into 3 stages based on imaging characteristics and observed that AUF begins with dorsal cortical thickening and progresses to a complete fracture. Furthermore, AUFs may, over time, become sclerotic and resemble pseudoarthrosis. In the treatment of AUF, a period of unloading and solid fixation should be considered according to the patient's walking condition.

## Data Availability

The data that support the findings of this study are not openly available due to privacy restrictions. The data that support the findings of this study are available from the corresponding author upon reasonable request.
